# Autoimmune encephalitis as a complication of COVID-19 infection: a case report

**DOI:** 10.1186/s43162-022-00119-7

**Published:** 2022-03-18

**Authors:** Ahmed Dahshan, Abeer Awad Abdellatef

**Affiliations:** 1 Department of Neurology, Kasr Al-Ainy School of Medicine, Cairo University, Cairo, Egypt; 2 Department of Internal medicine, Hepato-Gastroenterology Unit, Kasr Al-Ainy School of Medicine, Cairo University, Cairo, Egypt

**Keywords:** COVID-19 infection, Autoimmune encephalitis

## Abstract

**Background:**

During COVID-19 pandemic, a lot of newly discovered symptoms and presentations are emerging. Neurological symptoms of corona virus disease 19 (COVID19) have been reported including central nervous symptoms (CNS), peripheral nervous symptoms (PNS), and skeletal muscular symptoms; however, data are scarce about the exact occurrence of neurological affection during COVID-19 infection.

**Case presentation:**

We present a case of a 67-year-old male patient with proven COVID-19 infection who developed acute confusion state, behavioral changes, agitation, and one attack of loss of consciousness 8 days following the infection. Laboratory profile, computed tomography (CT) brain, magnetic resonance imaging (MRI), and cerebrospinal fluid (CSF) analysis all were normal, and the patients were highly suspicion of autoimmune encephalitis due to COVID-19 infection. The patient received pulse steroid therapy with complete regaining the conscious level.

**Conclusion:**

This clinical case emphasizes the possible relationship between COVID-19 infection and autoimmune encephalitis.

## Background

Being a global pandemic as the World Health Organisation (WHO) declared on March 11, 2020; COVID-19 infection presents with different presentations [1]. Neurological complication are among the COVID-19 presentations; however, the exact incidence of remains unknown [2].

Matching with the global changes in available data about COVID-19 infection and the huge efforts that exert in the research area, new emerging data is recording every second. During the first wave of COVID-19 infection, the neurological complications were observed mainly in severe COVID-19 infections, but recently the neurological complications could see in the early stages of the disease and even considers the second most common symptom after respiratory symptoms [3].

Neurological manifestations concomitant with COVID-19 infection can be categorized into three main categories; either central nervous symptoms (CNS), peripheral nervous symptoms (PNS), or skeletal muscular symptoms [4]. These clinical features may be the presenting complaints or maybe developed during illness [2].

## Case presentation

We report a case of a 67-year-old male patient. His past medical history was free apart from antihypertensive medications. His family history was negative for any neurological diseases. He was complaining of low-grade fever dry cough, sore throat, and loss of taste and smell for which the nasopharyngeal swab was done using real-time RT–PCR and was tested positive for COVID-19. He received home treatment in form of vitamins only. Eight days later, the patient started to develop acute confusion, behavioral changes, agitation, and one attack of loss of consciousness. Therefore, the patient was admitted to the hospital, the rest of the physical examination was unremarkable apart from elevated blood pressure up to 200/100. Chest, cardiac and abdominal examinations were clear. Neurological examination was positive only for disturbed conscious level and bilateral extensor planter, with normal tone and reflexes. Sensory, cranial nerves, and coordination examination could not be assessed. Fundus examination was normal. His laboratory profile was normal including a full complete blood picture (CBC), chemistry, thyroid profile, and serum electrolytes. Computed tomography (CT) brain and magnetic resonance imaging (MRI) was normal. Cerebrospinal fluid (CSF) analysis was done and was normal (normal cell count, normal proteins, glucose, LDH, and chloride); also, polymerase chain reaction (PCR) for HSV1, 2 were tested in the CSF analysis and was negative. Upon neurological consultation, and after well controlling to the blood pressure without any improvement to the patient condition, the patient received an intravenous (IV) pulse steroid in the dose of 1 mg/kg/day for 5 consecutive days. After the second dose of IV steroid, the patient regained full consciousness, without any residual behavioral changes or agitations. The patient was discharged from the hospital after full recovery without any residual neurological deficit.

## Discussion

The severe acute respiratory syndrome coronavirus-2 (SARS-CoV-2) infection could present with a wide range of manifestations that are not only restricted to respiratory symptoms. Several neurological complications concomitant with SARS-CoV-2 infection have been reported [5]; moreover, the SARS-CoV-2 is supposed to have a neuroinvasive potential similar to other viruses in the β-coronavirus group [6]; the exact explanation for that maybe related to direct viral effect, autoimmune mechanism, or may be a part of a paraneoplastic syndrome [7].

We reported a case of a 67-year-old male who developed mild COVID-19 and developed 8 days later concomitant autoimmune encephalitis. The interesting in our case is that the picture of encephalitis presented acutely and more than a week after the COVID-19 infection; hopefully, the patient responded rapidly to IV steroids that confirm our explanation for autoimmune theory.

Autoimmune encephalitis refers to a group of conditions that occur when the body’s immune system mistakenly attacks healthy brain cells, leading to inflammation of the brain. People with autoimmune encephalitis may have various neurologic and/or psychiatric symptoms. Neurologic symptoms may include impaired memory and cognition, abnormal movements, seizures, and/or problems with balance, speech, or vision. Psychiatric symptoms may include psychosis, aggression, inappropriate sexual behaviors, panic attacks, compulsive behaviors, euphoria, or fear. Symptoms may fluctuate, but often progress over days to a few weeks. Symptoms can progress to loss of consciousness or even coma [8].

Autoimmune encephalitis (AE) refers to a group of non-infectious immune-mediated inflammatory disorders involving mainly the cortical and deep grey matter with or without involvement of the white matter, meninges, or the spinal cord [9]. Diagnosis of autoimmune encephalitis can be made when all three of the following criteria have been met [10]:

1. Acute to subacute onset (rapid progression of less than 3 months) of working memory deficits (short-term memory loss), altered mental status, or psychiatric symptoms.
2. At least one of the following:
  New focal CNS findings
  Seizures not explained by a previously known seizure disorder
  CSF pleocytosis (white blood cell count of more than five cells per mm^3^)
  MRI features suggestive of encephalitis
3. Reasonable exclusion of alternative causes

Treatment may involve intravenous immunosuppressive therapy as intravenous methylprednisolone (IVMP) and early treatment decreases the likelihood for long-term complications, speeds recovery, and reduces the risk of recurrence (relapse) [11].

The high index suspicion of autoimmune encephalitis following COVID-19 infection should be raised in patients presenting with a combination of neurological and neuropsychiatric symptoms.

## Conclusion

The awareness should be raised to the possibility of post–COVID-19 autoimmune syndromes that could be occurred even after several days from complete resolution of infectious symptoms. Furthermore, the effects of COVID-19 on the nervous system and neurological outcomes after the infection is still not well studied; therefore, further studies are needed to clarify the potential relationship between COVID-19 and neurologic injury.

## Data Availability

Not applicable

## Abbreviations

SARS-CoV-2: Severe acute respiratory syndrome coronavirus-2
CT: Computed tomography
MRI: Magnetic resonance imaging
CSF: Cerebrospinal fluid
WHO: World Health Organization
CBC: Complete blood picture
IV: Intravenous
AE: Autoimmune encephalitis
IVMP: Intravenous methylprednisolone
